# Sexual dimorphism and population divergence in the Lake Tanganyika cichlid fish genus *Tropheus*

**DOI:** 10.1186/1742-9994-7-4

**Published:** 2010-01-22

**Authors:** Juergen Herler, Michaela Kerschbaumer, Philipp Mitteroecker, Lisbeth Postl, Christian Sturmbauer

**Affiliations:** 1 Institute of Zoology, University of Graz, Graz, Austria; 2 Department of Theoretical Biology, Faculty of Life Sciences, University of Vienna, Vienna, Austria

## Abstract

**Background:**

With about 120 colour morphs currently assigned to six nominal species, the genus *Tropheus *is an ideal model to study evolutionary divergence of populations in allopatry. The morphology of *Tropheus *has been described as relatively static, but reproductive constraints are sexually dimorphic due to mouthbrooding in females. We analysed phenotypic variation in six populations of *T. moorii *and one population of *T. polli *using geometric morphometrics to assess morphological differences among sexes in relation to the differentiation of populations and species.

**Results:**

The mean shapes differed significantly between sexes, populations, and species even though within-sex variation exceeded the divergence among populations. The first principal component of Procrustes shape coordinates revealed differences between populations and species in mouth position and ventral head shape. The second principal component reflected sex-specific shape differences, mainly comprising a relatively larger female viscerocranium and, in particular, a larger buccal area. While shape variation between populations and between sexes was primarily located in the cranial region, within-sex variation was relatively uniform across all landmarks.

**Conclusions:**

Deviations of the between-population and between-sex pattern of shape variation from that within sex indicate that the differences in head shape likely result from both adaptations to female mouthbrooding and population-specific foraging strategies.

## Background

Sexual dimorphism is a ubiquitous phenomenon in animal taxa. Hedrick and Temeles [1] discuss three main adaptive mechanisms favouring the evolution of sexual dimorphism: sexual selection, dimorphic niches, and food competition. Sexual selection typically acts on males, e.g., when females show assortative mating or when mate competition enforces selection on certain male traits. In contrast, the dimorphic niche hypothesis suggests selection acting mainly on females, due to reproductive constraints [1]. Alternatively, sexual dimorphism can evolve by ecological selection acting differentially on both sexes and thus favouring both dimorphic niches and, as a consequence, dimorphic trophic structures [1]. Bolnick and Doebeli [2] found that sexual dimorphism usually evolves more rapidly than new species by disruptive ecological selection [3,4]. Intense interspecific competition, in contrast, is likely to reduce sexual dimorphism because of a restricted ecological niche for both sexes [1,5,6]. For example, Butler et al. [7] found that sexual dimorphism in lizards was greater in species-poor environments with reduced interspecific competition as compared to environments inhabited by more species.

The majority of studies on sexual dimorphism focused on humans, apes, birds, amphibians and fishes, and usually dealt with sexual size dimorphism (SSD) as summarized by Blanckenhorn [8]. In particular, many teleost fishes represent interesting models for studying sexual dimorphism [9], but few authors have addressed actual shape dimorphism, especially in relation to reproductive behaviour [10]. A range of sexually dimorphic reproductive constraints on shape have been reported for the cranial anatomy of mouthbrooding fishes, which incubate their eggs in the buccal cavity until the larvae hatch [e.g., [10]]. Oliveira and Almada [11] found that in maternally mouthbrooding cichlids of the Great East African Lakes, females have wider premaxillae, longer snouts, and larger preopercular and interopercular bones than males of these species. Disproportionate investment in reproduction by maternal mouthbrooders was suggested to result in strong sexual selection and thus often drives sexual dimorphism [12].

Our study populations of the genus *Tropheus *live in the East African Lake Tanganyika and are brightly coloured female mouthbrooders. Unlike most other haplochromine cichlids, they display no sexual dichromatism. The resemblance in colour patterns among sexes has been attributed to their function in communication within a territorial social system including males and females [13,14]. Although the overall morphology of populations and sister species of the genus *Tropheus *was considered as relatively static [15] except for colouration, which is highly polymorphic across geographically isolated populations, more recent investigations found subtle differences in trophic and head morphology [16,17]. About 120 colour morphs were described [18]. Most populations live in allopatry but in some locations two *Tropheus *populations live in sympatry (e.g., *T. moorii *and *T. polli *occur together on the east coast of Lake Tanganyika at Kekese; Sturmbauer personal observations). Although *Tropheus *populations have been subject to molecular phylogenetic and phylogeographic studies, we still lack comprehensive morphological investigations based on a large number of specimens and populations to quantify shape variation, population divergence, and sex-related differences within and among populations and species. Moreover, the genus is in need of taxonomic revision, as the six nominal species described so far do not include a sufficient number of populations and none of the recent molecular genetic results [19,20].

The present study investigates phenotypic divergence and sexual dimorphism in seven *Tropheus *populations from different regions of Lake Tanganyika. We quantified and visualized mean shape differences between sexes (sexual dimorphism), and between populations and species (morphological divergence) using a geometric morphometric approach. Because of the common mode of reproduction (mouthbrooding) in all populations, we hypothesized a similar pattern of sexual dimorphism, involving differences in the morphology of the oral cavity. We further compared the *amount *of sexual shape dimorphism among the populations, which is expected to inversely relate to the degree of interspecific competition. In order to assess whether morphological differences between populations and sexes can be traced back to selective forces, we visualized and compared the corresponding variance-covariance structures between populations, between sexes, and within sexes. Deviations between these matrices may indicate evolutionary scenarios involving directional or stabilizing selection.

## Methods

### Study populations

**Figure 1:**
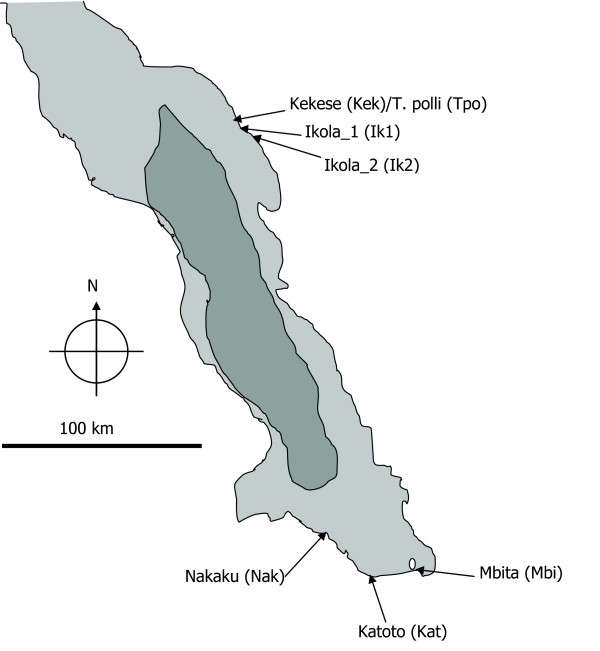
**Study populations of *Tropheus *from Lake Tanganyika**. The map shows the locations of six study populations of *Tropheus moorii *and one of *T. polli *(including abbreviations) from the eastern and southern part of Lake Tanganyika.

We examined seven populations of *Tropheus*, yielding a total sample of 691 specimens. Six populations of *Tropheus moorii *were sampled (Fig. 1): Kekese (126 males, 125 females), Ikola_1 (29 males, 57 females), Ikola_2 (56 males, 106 females), Katoto (17 males, 22 females), Mbita (22 males, 25 females) and Nakaku (25 males, 25 females). One population of *T. polli *(30 males, 26 females) was sampled at Kekese (living in sympatry with *T. moorii*). In the following text, the six *T. moorii *populations are named after their sampling location (Kekese = Kek, Ik1 = Ikola_1, Ik2 = Ikola_2, Katoto = Kat, Mbita = Mbi, Nakaku = Nak), whereas *Tropheus polli *from Kekese is abbreviated with "Tpo". Adult fish of all populations were used in this study to minimise shape variation due to ontogenetic allometry within each population. Populations were sampled across different spatial scales of allopatric distribution and habitats. *Tropheus polli *was chosen for interspecific comparison because it is a distinct species, which occurs sympatrically with three different populations of *T. moorii *at several locations without interbreeding. The two species also refused to hybridize under experimental conditions (Sturmbauer, unpublished data). In addition, the potential effect of interspecific competition on trophic divergence was studied by sampling two genetically close populations of *T. moorii*, one in sympatry and the second one in allopatry with *T. polli*.

### Data acquisition

**Figure 2:**
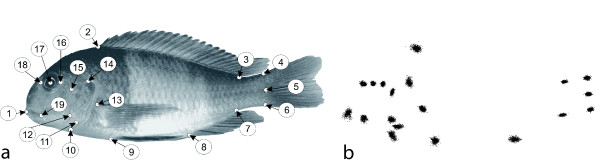
**Landmark dataset**. (a) One specimen of *Tropheus *with the 19 landmarks used for the geometric morphometric analysis (see Table 1 for definitions), b: Scatter plot of all 691 landmark configurations after Procrustes superimposition.

Fish were narcotised for 2 to 3 min with a low dose of clove oil (1-2 drops per liter water) and scanned on a specifically adapted flatbed scanner following the protocol of Herler et al. [21]. The sequence of digital images was randomized with TPSUtil 1.33 [22] and 19 landmarks (Table 1, Fig. 2a) were digitized on every specimen using TPSDig 2.10 [23].

**Table 1:** Definition of landmarks. Nineteen landmarks were digitized on 691 specimens of *Tropheus *(see also Fig. 2).

Landmark	Definition
1	anterior tip of snout
2	anterior insertion of dorsal fin
3	posterior insertion of dorsal fin
4	upper insertion of caudal fin
5	midpoint of the origin of caudal fin
6	lower insertion of caudal fin
7	posterior insertion of anal fin
8	anterior insertion of anal fin
9	insertion of ventral fin
10	anteroventral tip of pectoral girdle (cleithrum)
11	ventral-most point of the border between interopercle and subopercle
12	the point where preopercle, interoperculum and subopercle meet
13	upper insertion of pelvic fin
14	dorsal origin of operculum
15	dorsal end of preopercular groove
16	anterior rim of orbit along the horizontal body axis
17	centre of orbit
18	posterior rim of orbit along the horizontal body axis
19	posterior-most point of lips

### Data analysis

The 691 landmark configurations were superimposed by a Generalized Procrustes Analysis (Fig. 2b) and projected into tangent space [24,25]. The distribution of sex-specific mean shapes in shape space was evaluated with Principal Component Analysis (PCA), also called Relative Warp Analysis when applied to Procrustes shape coordinates [26]. The shape deformations depicted by the principal components (relative warps) are visualized by thin-plate spline (TPS) deformation grids [26], superimposed on images of a fish deformed by the same TPS functions (the fish image was selected to be close to the overall mean shape of all populations). Differences between mean shapes were tested for statistical significance with Monte-Carlo permutation tests [27].

In the style of a two factor nested multivariate analysis of variance, we decomposed the total variation of Procrustes shape coordinates into three components: (1) variation among population means, (2) variation among sex means (within each population), and (3) residual variation (within each population and sex). We visualized these three components of variance with scatter plots of the corresponding Procrustes coordinates and equal frequency ellipses for each landmark. We further calculated the corresponding sum of squares and variances: sum of squared deviations (SS) of the seven population means from the grand mean, SS of the 14 sex means from the corresponding population means, SS of each individual from the corresponding sex mean. See the Appendix for more details and an alternative analysis.

According to Lande [28], the genetic between-population covariance matrix (i.e., additive and heritable phenotypic variance and covariance) is expected to be proportional to the within-population covariance matrix under pure genetic drift. Deviations from proportionality may indicate evolutionary scenarios involving directed or stabilising selective forces. Heritable between-sex variation is additionally constrained by genetic correlations between homologous characters in males and females [5]. The 'graphical decomposition' of variance as described above allows a visual inspection and localisation of differences in the patterns of phenotypic variance and covariance. We also performed likelihood ratio tests of homogeneity and proportionality for the three covariance matrices [29]. All statistical and morphometric analyses were performed in Mathematica 6 using routines programmed by Philipp Mitteroecker and Philipp Gunz.

## Results

**Figure 3:**
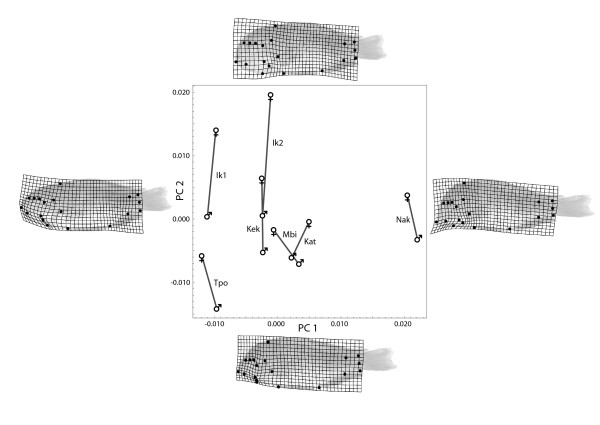
**Principal component analysis of shape coordinates**. Scatter plot of the first two principal component scores for the 14 sex-specific mean shapes. The two mean shapes for each population are connected by a grey line to indicate the direction of sexual dimorphism. The deformation grids on the left and the right side visualize the shape change depicted by PC 1; they are deformations of the mean shape to shapes corresponding to minus and plus 0.07 units along the PC 1 axis. Similarly, the top and bottom deformation grids visualize shape change along PC 2. See the Methods section for abbreviations of populations.

Permutation tests indicated that the mean shapes of each *Tropheus *population differed significantly from each other (*P *< 0.001 for all 28 tests, 5,000 permutations). Pairwise linear discriminant analyses even demonstrated that most groups have little or no overlap in shape space (results not shown). Furthermore, male and female mean shapes differed significantly within all seven populations (*P *< 0.008). Figure 3 shows the first two axes of a principal component analysis (PCA) of the fourteen sex-specific mean shapes (explaining 87% of total variation among the mean shapes). While the seven populations differed along both the first and the second PCs, shape differences between sexes were largely confined to the second dimension. Population differences along the first PC were mainly based on the shape (jaw retraction or protrusion) and the relative position of the mouth (terminal or subterminal), where *T. moorii *from Nakaku on the one hand, and *T. polli *and *T. moorii *Ikola_1 on the other hand represent the opposite extremes. The remaining four populations of *T. moorii *exhibited less shape variation in this dimension; especially Katoto and Mbita males were almost indistinguishable. Minor shape differences associated with PC 1 were also located in the caudal peduncle.

A common direction of sexual dimorphism was evident in shape space (predominately along PC 2), although the exact orientation and degree of sexual dimorphism varied across the seven surveyed populations (Fig. 3). A permutation test rejected the null hypothesis of equal degrees of sexual dimorphism (Procrustes distance between male and female mean shapes) in all populations with *P *< 0.001. The two populations near Ikola (Ikola_1 and Ikola_2) exhibited the largest sexual dimorphism in body shape, whereas especially the southern populations at Katoto, Mbita and Nakaku were less dimorphic. Females had a relatively larger head than males, particularly a larger ventral area (buccal region). Especially the area between the landmarks of the posterior edge of the mouth gape, opercular series and the anteroventral tip of the cleithrum (pectoral girdle) - the characters representing the external markers for the buccal cavity - exhibited strong local shape deformations.

**Figure 4:**
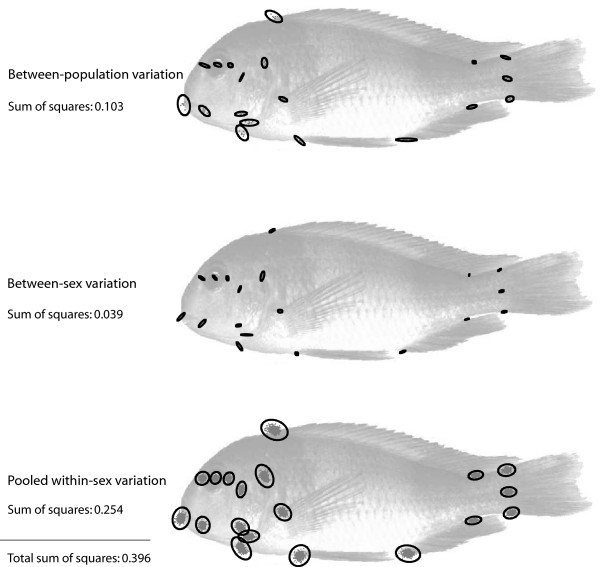
**Decomposition of shape variation**. The total variation of Procrustes shape coordinates (Fig. 2b) was decomposed into three components: the variation between population mean shapes, the variation between sex-specific mean shapes within populations, and the pooled residual variation within sexes and within populations. The figure shows the landmark scatters with equal frequency ellipses for these three components along with the corresponding sum of squares. The variances of these components are 2.423, 0.512, and 3.681, respectively (summed over all landmarks and multiplied by 10^4^).

Figure 4 shows the decomposition of the total variation of Procrustes coordinates (as depicted in Fig. 2b) into three components: the variation among population mean shapes, the variation among sex-specific mean shapes (within populations), and the residual variation within sex and within population (ignoring variation between sexes and populations). Likelihood ratio tests yielded significant differences among the three respective covariance matrices: they were neither identical nor proportional. As visualised by the three separate landmark scatters, there was more shape variance (summed over all landmarks and multiplied by 10^4^) within one sex (3.681) than between sexes (0.512) and between populations (2.423). Whereas the amount of within-sex variation was somewhat similar for all landmarks, the cranial and particularly the oral landmarks exhibited considerably more between-sex as well as between-population variation than the other landmarks. For the residual within-sex variation, the average variance per cranial landmark was approximately 0.84 times the average postcranial variance. For between-population and between-sex variation, this ratio was 1.30 and 1.84, respectively.

## Discussion

A wide range of forms and sexual dimorphism patterns has evolved in teleost fishes when compared to other vertebrates. In the family Cichlidae most studies focused on sexual size dimorphism (SSD) [30,31] and only few authors addressed shape dimorphism with respect to reproductive behaviour [e.g., [32]]. While SSD can be quantified by simple measures of overall size, studies of shape dimorphism require a series of measured characters. Traditional multivariate morphometric approaches are typically based on measures such as Euclidian distances, areas, or counts. Differences in shape are usually inferred from disproportional changes of these single size measures across individuals or group means, but the actual geometry underlying these changes cannot be reconstructed from such measurements. Geometric morphometrics, in contrast, is based on landmark coordinates, allowing statistical results to be represented as actual shapes or shape deformations. Due to the increased number of variables in most geometric morphometric studies, shape differences can be assessed in more detail and exploratory studies can be performed more successfully [17,26,33-35].

Sexual selection was argued to play a role in the explosive speciation and adaptive radiation of cichlid fishes [36-39] although its actual role in the genus *Tropheus *remains controversial, as the genus does not display sexual dichromatism [40,41]. Danley and Kocher [42] stated that morphological adaptations to differential food niches occurred at an early stage of the radiation. A recent study empirically supported the hypothesis that divergent natural selection has shaped trophic morphology [43]. Due to its old evolutionary age, the cichlid fish species flock of Lake Tanganyika is at a mature stage of adaptive radiation, in which species are eco-morphologically highly diverse and live in complex communities so that diversification has slowed down [39]. Stabilizing selection is thought to prevail in such a mature species community. Remarkably, many littoral species show considerable geographic variation, mostly in terms of colour. Populations of the genus *Tropheus *were previously thought to be morphologically largely invariant and differentiated mainly by colour in allopatric populations [15,18,44]. Maderbacher et al. [17] and Postl et al. [16], however, discovered slight, yet significant, morphological differences among three populations of *Tropheus*.

The present study demonstrated considerable morphological variation among the six populations of *T. moorii *and one population of *T. polli *from Lake Tanganyika, even though within-population variation exceeded the variation among populations. The main differences between population mean shapes were in overall body shape and particularly in the landmarks defining the shape (retracted versus protruded) and position (terminal versus subterminal) of the mouth and the lateral outline of the buccal cavity. Similarly, Kassam et al. [32] reported interspecific differences of mouth length in three congeneric species of the cichlid genus *Petrotilapia *from Laka Malawi, in which trophic niche partitioning was assumed to be the main driving force for morphological divergence.

We also found among population variation of landmarks at the dorsal and anal fin origins, which may be explained by population-specific differences in the number of fin spines. While *T. polli *usually has four anal spines, *T. moorii *populations at Ikola and Kekese have five and those at Nakaku, Mbita and Katoto have six [[18,45], personal observations]. In contrast, the number of dorsal spines is more conserved (fish from Ikola had 20, whereas all other populations had 21). Minor shape differences were present in the posterior trunk region, particularly in the caudal peduncle shape; the latter is assumed to affect swimming type and performance, and consequently foraging strategy. A longer caudal peduncle often is associated with prolonged swimming while a deeper peduncle characterizes more powerful sprint swimming [46]. Male *Tropheus *had a larger caudal peduncle area than females, and the population from Nakaku had a larger area than those from Ikola or *T. polli*. This may assist in burst swimming for territorial defence, while the (fin) shape differences between populations potentially indicate different foraging strategies or predation pressure.

The Nakaku population was most distinct in shape, whereas the eastern populations of *T. moorii *were even more similar to *T. polli *than to their conspecific populations from the southwest. The Nakaku population belongs to a different major mtDNA lineage of *T. moorii*, the so-called Chaitika lineage, and is also distinct in colour [20,44]. Interestingly, *T. moorii *and *T. polli *from Kekese live in sympatry and show morphological differences in the mouth region (see Fig. 3), indicating a trophic niche segregation. Similarly, Kassam et al. [32] reported slight differences in mouth morphology of co-existing species of the genus *Petrotilapia *from Lake Malawi, suggesting partitioning of food resources. At Kekese, *T. moorii *lives in deeper water, probably because of competition with *T. polli*, which grows larger and occupies the most shallow water zone. This zone is usually also occupied by *T. moorii *in other locations throughout the lake (Sturmbauer, personal observations). Slight habitat differences (e.g. in terms of wave action) and different species and abundances of epilithic algae - the main food source of *Tropheus *- exist in deeper water, so that a different position and protrusion of the mouth might reflect adaptations to these conditions. The adjacent populations of *T. moorii *near Ikola (Ikola_1 and Ikola_2) belong to the same colour morph as fish from Kekese, but live without *T. polli*. They also occupy the uppermost littoral zone. Fish from Ikola_2 were morphologically indistinguishable from those at Kekese within the first two principal components, whereas fish from Ikola_1 were more distant from the other two conspecific populations, but highly similar to the allopatric *T. polli*, particularly in the terminal mouth; the latter may represent a case of convergence. Thus, among these eastern populations, allopatric populations were more similar to each other than those living in sympatry. In contrast, the morphological similarity of the southern populations at Mbita and Katoto might reflect their close phylogenetic relationship and recent gene flow. These two populations are also similar in colour and mitochondrial DNA, suggesting a recent admixture of the Katoto population [20].

Our analysis indicates that ecological divergence has contributed to the diversification of the genus *Tropheus*. Stronger evidence, however, requires an eco-morphological study based on a larger number of populations and more precise ecological data. In addition to ecological factors, positive assortative female mate preference, which has led to about 120 different colour morphs, has probably played a major role in at least an allopatric diversification process [36,41], whereas some studies questioned the importance of (colour)assortative mating for sympatric speciation in *Tropheus *[[40], Egger et al. unpublished].

The PCA (Fig. 3) showed a common pattern of sexual dimorphism in all seven populations along the second principal component. This component mainly consisted of a dorso-ventrally expanded head, an extended buccal region, and a slight contraction in the ventral opercular region in females. These sex-specific morphological differences relate to the reproductive constraints on the cranial anatomy of mouthbrooding fishes. Oliveira and Almada [11] reported sexual dimorphism in jaws and fins of other maternally mouth-brooding cichlids and related these differences mainly to sexual selection, while Kassam et al. [32] did not detect any sexual dimorphism in rock-dwelling cichlids of the genus *Petrotilapia *from Lake Malawi. Barnett and Bellwood [10] reported a significantly larger buccal cavity in males of the paternally mouthbrooding Apogonidae, where the degree of dimorphism varies across species. Furthermore, they found that these shape differences were not only reflected in the osteology, but also in soft tissue morphology. The landmarks used in the present study mainly represent bone morphology, but it is very likely that soft tissue flexibility contributes to the sexual dimorphism in *Tropheus *as well.

Even though the pattern of sexual shape dimorphism was similar in all seven populations, they differed considerably in the magnitude of sexual dimorphism. There were less sex-specific shape differences within the three southern populations as well as in *T. polli *than in the more dimorphic eastern populations of *T. moorii *near Ikola. In the closely related and morphologically similar populations at Mbita and Katoto, sexual dimorphism even exceeded the morphological differences at the population level. Interestingly, sexual dimorphism in these two populations was not only found along the second principal component, but the females also differed along the first principal component from the males, indicating that also additional ecological selective forces may have contributed to sexual dimorphism. Although our study presents only a single case of interspecific competition in sympatry (that of *T. polli *and *T. moorii *at Kekese), which is insufficient to infer a general pattern, the finding of lower sexual dimorphism in *T. moorii *from Kekese when compared to Ikola (Fig. 3) is congruent with the hypothesis of an inverse relationship of sexual dimorphism and interspecific competition [1,6,7]. Additionally, variation in the magnitude of sexual dimorphism may relate to population differences in the size and number of eggs stored by females, but we lack such data for our populations.

In a decomposition of shape variation (Fig. 4) we found that between-population and between-sex variation was mainly located in the cranial region, whereas within-sex variation was relatively uniform across all parts of the body. Especially the between-population variation is largely confined to the mouth and viscerocranium. In the case of pure evolutionary drift, the genetic between-population covariance structure (the additive and heritable part of phenotypic variation) is expected to be proportional to the variance and covariance within populations. Deviations from that pattern are likely due to directional or stabilising selection [28,47]. Variation between sexes is further determined by genetic correlations between male and female traits [5]. Of course, our decomposition of phenotypic variance is not a rigorous test of selection; instead, it permits an exploration of gross differences between patterns of phenotypic variation. The highly localised variation among populations and between sexes relative to the uniform within-sex variation indicates that both sexual dimorphism and population divergence are due (partly) to selection. The sexually dimorphic cranial morphology in the mouthbrooding cichlids may be explained as an evolutionary adaptation to the dimorphic reproductive regimes. Among-population (and interspecific) variation in cranial morphology may reflect adaptations to different habitats and foraging strategies.

## Conclusions

By the application of geometric morphometrics, we demonstrated that populations of *T. moorii *and *T. polli *in Lake Tanganyika have evolved subtle morphological differences. Sex-related differences are mainly evident in the larger buccal area of females and can be explained as adaptation to maternal mouthbrooding. In contrast, population-specific differences mainly involve the position of the mouth, which may be a result of different trophic ecological selection regimes in different habitats. Further sex- and population-specific differences are located in the caudal peduncle, indicating different locomotion patterns during feeding and territorial behaviour of males. The observed variation in the magnitude of sexual dimorphism indicates an inverse relationship of sexual dimorphism and interspecific competition.

## Appendix

In the main text, we decomposed the total variation of Procrustes shape coordinates into three components: the variation of population means around the grand mean, the variation of sex means (within each population) around their corresponding population mean, and the residual variation of individuals around their corresponding sex mean. This is analogous to a so-called nested or hierarchical design in an analysis of variance (ANOVA). An alternative approach would be a two-way ANOVA leading to four components of variation: two factors, an interaction term, and the residuals. Two-way ANOVA is applied when two factors of equal rank have approximately independent effects on the individuals. A nested design is typically used when the main group (main factor) is subdivided into randomly chosen subgroups (the nested factor), which may thus not be the same for all main groups [48]. As the correct design for our data is not obvious we discuss our choice here and present an alternative analysis.

In our case, sex (as the nested factor) apparently has the same levels (male/female) in each species, which are not chosen randomly. On the other hand, they represent *all *possible levels of the sex factor, and it appears natural to consider sex as nested within population or species rather than of the same rank as population/species. We thus decided to apply a nested design to visualize how sexual dimorphism *varies *across the seven populations/species, relative to the variation within the sexes and between the populations. In other words, our primary interest in this analysis of variance was not the average effect of being male or female, but how sexual dimorphism varies across the seven populations.

Under the (apparently unrealistic) null-hypothesis of sexual dimorphism due to random drift only, dimorphism would have evolved independently within the populations and would not share a common (selective) factor across all populations. In fact, we would expect a distribution resembling the within-sex distribution, modified by genetic correlations between the sexes. We found that the distribution of sex means clearly differs from the within-sex variation, which was nearly isotropic. Instead there was far more variation between sexes in the head and particularly in the buccal area than a scenario of pure drift would lead us to expect. As mentioned in the main text, this is not a formal test of selection versus drift, but rather an exploratory approach to visualize different aspects of phenotypic shape variation.

**Figure 5:**
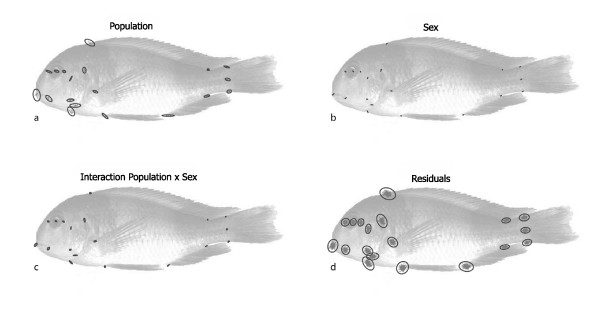
**Decomposition of shape variation in the style of a two-way ANOVA**. The total variation of Procrustes shape coordinates (Fig. 2b) was decomposed into four components: variation of population mean shapes, the variation of sex mean shapes, variation due to the interaction population x sex, and the residual variation.

In Figure 5, we analyzed the same data with a two-way ANOVA instead of a nested design. The distribution of the seven species means (Fig. 5a) is the same as in Figure 4, but sex as the second main factor differs. It has two levels only, which are visualized in Figure 5b as the difference between average male and average female landmark positions. Similar to PC 2 in Figure 3, it shows that the average effect of sexual dimorphism is mainly located in the head. A likelihood ratio test indicates that the interaction term differs significantly from zero (*P *< 0.001). The interaction term (Fig. 5c) depicts how the actual 14 sex means deviate from their expectations derived from the *average *sex and population effects. In other words, it shows how sexual dimorphism varies across the populations, independent of the average sexual dimorphism. Again, this variation is largely constrained to the cranium and the buccal area. The residual term (Fig. 5d) is the same for both types of analyses.

The two-way ANOVA leads to the same interpretation of the data as the nested ANOVA, but it is unclear whether a decomposition of sexual dimorphism into an average effect and an interaction term is more natural than a single term for the variation between sex means as in the nested design. Computationally, both approaches are feasible, particularly as the sums of squares and significance levels are of minor interest in this exploratory attempt. Their usefulness primarily depends on the scientific context.

## Competing interests

The authors declare that they have no competing interests.

## Authors' contributions

JH and CS designed the study. JH took the leading role in working on the manuscript. MK, LP and CS sampled fish, MK digitized the landmarks, and all three persons contributed to the manuscript. PM performed the statistical analysis, designed the figures, and made important contributions to the manuscript. All authors read and approved the final version of the manuscript.
